# The DYW Subgroup PPR Protein MEF35 Targets RNA Editing Sites in the Mitochondrial *rpl16*, *nad4* and *cob* mRNAs in *Arabidopsis thaliana*

**DOI:** 10.1371/journal.pone.0140680

**Published:** 2015-10-15

**Authors:** Nadja Brehme, Eszter Bayer-Császár, Franziska Glass, Mizuki Takenaka

**Affiliations:** Molekulare Botanik, Universität Ulm, Ulm, Germany; National University of Rosario, ARGENTINA

## Abstract

RNA editing in plant mitochondria and plastids alters specific nucleotides from cytidine (C) to uridine (U) mostly in mRNAs. A number of PLS-class PPR proteins have been characterized as RNA recognition factors for specific RNA editing sites, all containing a C-terminal extension, the E domain, and some an additional DYW domain, named after the characteristic C-terminal amino acid triplet of this domain. Presently the recognition factors for more than 300 mitochondrial editing sites are still unidentified. In order to characterize these missing factors, the recently proposed computational prediction tool could be of use to assign target RNA editing sites to PPR proteins of yet unknown function. Using this target prediction approach we identified the nuclear gene *MEF35* (*Mitochondrial Editing Factor 35*) to be required for RNA editing at three sites in mitochondria of *Arabidopsis thaliana*. The MEF35 protein contains eleven PPR repeats and E and DYW extensions at the C-terminus. Two T-DNA insertion mutants, one inserted just upstream and the other inside the reading frame encoding the DYW domain, show loss of editing at a site in each of the mRNAs for protein 16 in the large ribosomal subunit (site *rpl16*-209), for cytochrome b (*cob*-286) and for subunit 4 of complex I (*nad4*-1373), respectively. Editing is restored upon introduction of the wild type *MEF35* gene in the reading frame mutant. The MEF35 protein interacts in Y2H assays with the mitochondrial MORF1 and MORF8 proteins, mutation of the latter also influences editing at two of the three MEF35 target sites. Homozygous mutant plants develop indistinguishably from wild type plants, although the RPL16 and COB/CYTB proteins are essential and the amino acids encoded after the editing events are conserved in most plant species. These results demonstrate the feasibility of the computational target prediction to screen for target RNA editing sites of E domain containing PLS-class PPR proteins.

## Introduction

RNA editing sites in mitochondria and in plastids in flowering plants are recognized by PPR proteins (Pentatrico-Peptide-Repeat Proteins), a family of about 450 nuclear encoded proteins [1–4]. The composition of the variable number and sequence of the PPR elements of about 35 amino acids each determine the target RNA sequence motif to which these proteins reversibly attach. Consequently, each of these proteins is required for one or several specific RNA editing events that have to be identified experimentally. A number of such assignments have been resolved for plastid as well as for mitochondrial PPR proteins, which allowed to deduce the parameters in the PPR elements which determine the sequence specificity. A combination of amino acids at two (or three) specific positions of each PPR element determine the nucleotide identity to be selected [5–9]. This PPR—RNA code is supported by the crystal structure analysis of a non-editing PPR protein, PPR10 [10–13]. PPR10 is one of the about 250 PPR proteins with canonical 35 amino acids long repeats, while all identified PPR proteins involved in RNA editing are composed of variations of 35 amino acids elements (P), shorter (S) and longer ones (L), the PLS-class.

The RNA editing PPR proteins are also characterized by additional C-terminal domains, all containing at least one extension domain (E domain) and about half of them being further extended by the so-called DYW domain [14–21]. The designation of the DYW extension derives from the often found C-terminal amino acid triplet DYW. In addition to the E domain containing PPR proteins, MORF proteins [22] are required for numerous editing sites (MORF = multiple site organellar RNA editing factors). The precise function of the MORF proteins still has to be elucidated in detail.

In flowering plants, about 90 E subgroup and 100 DYW subgroup PLS class PPR proteins are encoded, which contain only E or E and DYW domains, respectively. Most of the characterized E or DYW subgroup PPR proteins are involved in RNA editing, the rest of them play a role in other RNA processing steps. For example, the proteins OTP70 and PpPPR_43 are involved in intron splicing and CRR2 is involved in intercistronic RNA cleavage in plant organelles [23–25]. Direct experimental analysis of all RNA editing sites to determine the function of a given E domain containing PPR protein is straightforward but bears the risk of analyzing proteins not involved in RNA editing. Therefore, computational prediction for target RNA binding sites of each PLS class PPR protein could be helpful in the first screen for RNA editing factors from the E domain containing PPR proteins. Investigating and evaluating the feasibility of this approach, we here report the computational prediction based characterization of site-specific RNA editing trans-factor, MEF35, which is required for three editing events, one each in the *rpl16*, *nad4* and *cob* mRNAs in mitochondria of *Arabidopsis thaliana*.

## Results

### Identification of the RNA editing target sites in mutant plants of At4g14050

**Fig 1:**
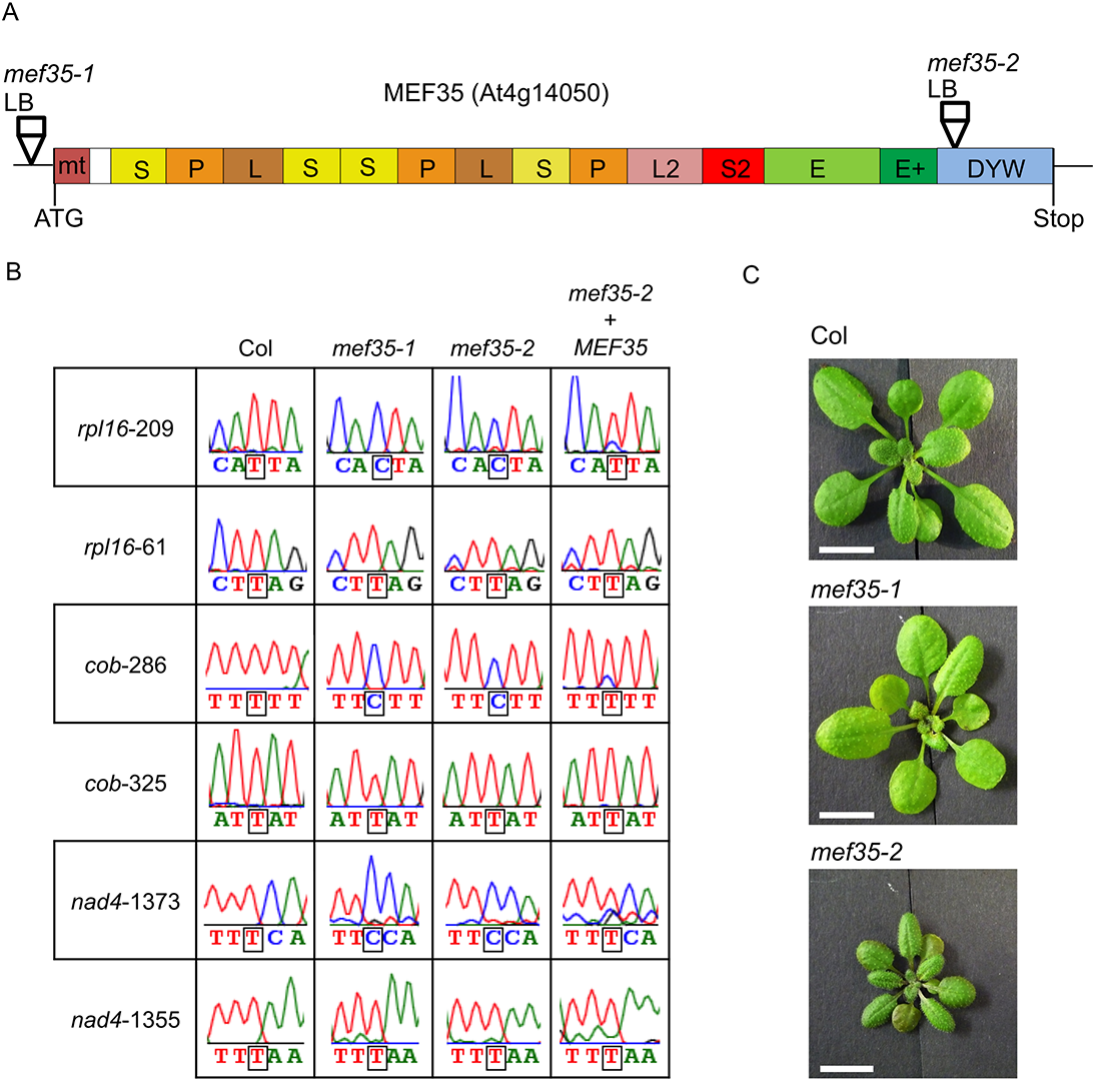
The MEF35 protein is required for RNA editing at the *rpl16*-209, *nad4*-1373 and *cob*-286 editing sites in mitochondria of *Arabidopsis thaliana*. **(A)** Schematic structure of the MEF35 PPR-protein encoded by locus At4g14050. Locations of the T-DNA insertions in the two mutants and their respective left borders (LB) are indicated. The predicted types of PPR elements, P, L or S, as well as the E and DYW motifs are labelled. The region marked E+ is also considered part of the E domain. **(B)** Analysis of editing in *mef35-1* and *mef35-2* mutant plants. Comparison of the cDNA sequence analysis of three RNA editing sites (boxed) in the mitochondrial *rpl16*, *nad4* and *cob* mRNAs between wild type *Arabidopsis thaliana* (wt) and the *mef35-1* and *mef35-2* mutant plants shows that both mutants have lost the ability of C to U editing at these sites. Three other editing sites in the respective same mRNAs are shown as controls, these sites are correctly edited in wild type and both mutant plants. In the cDNA strands analysed, the detected T nucleotide (red trace) corresponds to the edited U, the observed C (blue trace) is derived from an unedited C. The right hand analyses (*mef35-2+MEF35*) show that the Col *MEF35* gene sequence restores the ability for RNA editing in transgenic plants of mutant line *mef35-2*. (C) The plants of mutant line *mef35-1* show developmental and adult phenotypes indistinguishable from the wild type. Seedlings of mutant line *mef35-2* initially develop a little slower in comparison to the wild type Col plantlets, but look similar at the flowering stage (S1 Fig). Transgenic complemented plants of *mef35-2* also display the retarded growth indicating that this phenotype is not related to the *mef35-2* T-DNA insertion. Four weeks old plants grown side by side are shown.

The functional connection identified between specific RNA editing sites and individual PPR proteins suggests that a large number of these proteins will be involved in the editing of one or several editing sites. To further identify specific PPR proteins connected to individual RNA editing sites in plant mitochondria, we selected the gene in locus At4g14050 in *Arabidopsis thaliana* (Fig 1A). This gene codes for a DYW containing PLS-class PPR protein and is predicted to be imported into mitochondria by two different target prediction programs, targetP ([26] and Predotar ([27]. Two T-DNA insertion lines in the At4g14050 locus, *mef35-1* and *mef35-2* with an insertion upstream and inside the reading frame encoding the DYW domain, respectively, were genotyped and homozygous plants for the respective alleles were obtained (Fig 1A).

**Fig 2:**
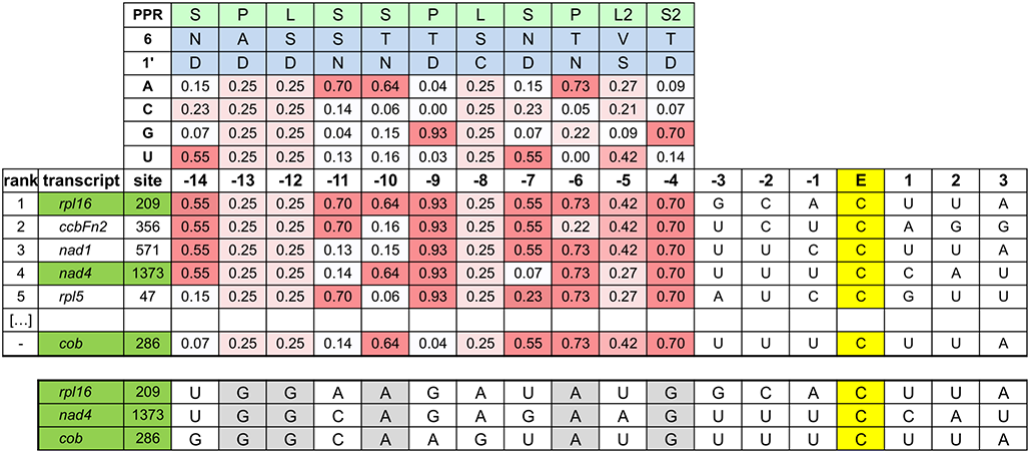
Prediction of the target sites of the mitochondrial RNA editing factor MEF35. The prediction tool derived from nucleotide–amino acid coincidences between a given PPR element and the corresponding nucleotide was used to predict and rank editing targets for MEF35 by probability [8]. In this listing, the observed target editing sites *rpl16*-209 and *nad4*-1373 are highlighted. The third site *cob*-286 was not predicted by this approach. The putative binding sequences are aligned and the PPR scores are given. These indicate less intensive binding in the N-terminal elements. As generally assumed, alignment begins at nucleotide -4 from the edited C (marked E; [5–9]. The nucleotide sequences of the three bona fide target sequences are shown in the bottom part.

To identify the potential RNA editing target sites of At4g14050, we applied the recently established computational prediction method which has been shown to predict target sites of known PPR RNA editing factors with reasonable success [8] (Fig 2). At first we sequenced the top 10 predicted candidate sites in the cDNAs of the two mutant lines of At4g14050. Both T-DNA insertion lines show a loss of RNA editing at *rpl16*-209 and *nad4*-1373, which are ranked 1st and 4th in the prediction, respectively (Fig 1B). The other highly ranked sites and those editing sites contained in the analysed cDNAs were all edited as in Col wild type plants. To evaluate the precision of the prediction tool, we analysed all other editing sites in mitochondrial open reading frames by direct sequence analysis and identified a third site to be affected in the mutants, nucleotide *cob*-286 (Fig 1B). No other editing sites in mitochondrial open reading frames are affected in the mutant plants.

### The intact *MEF35* gene recovers RNA editing in mutant plants

To confirm the direct involvement of the *MEF35* gene in these RNA editing events, we made stable transformants with the reading frame encoded by the At4g14050 locus under the 35S promoter in *mef35-2* mutant plants. In these transgenic plants, RNA editing was restored at sites *rpl16*-209, *cob*-286 and *nad4*-1373 to the levels observed in Col wild type plants (Fig 1B, *mef35-2*+*MEF35*). The gene encoded by At4g14050 was thus confirmed as the specific RNA editing factor for these sites and we renamed this gene to code for mitochondrial editing factor 35, MEF35.

### Loss of RNA editing at the target sites in MEF35 mutant plants does not affect growth

Initially, *mef35-2* young mutant plantlets develop somewhat slower than the wild type (Fig 1C) and also than the *mef35-1* mutant plantlets, but stem and flower development look identical (S1 Fig). To determine whether this effect is caused by the *mef35-2* mutation, we selected this line for the stable transformation with the wild type reading frame. Complemented plants show the same phenotype with an initial developmental delay, confirming that this is most likely due to other T-DNA insertion or mutation events in the genome. This conclusion is also supported by the entirely normal phenotypic development and growth of the other mutant, the *mef35-1* mutant plants.

The wild type-like growth pattern of *mef35-1* mutant plants is surprising, considering that the affected editing events exchange highly conserved amino acids in the RPL16 (I>T) and in the apocytochrome b proteins (F>L). Whether the function of the ribosomal complex is affected by the amino acid alteration in subunit 16 of the large subunit is discussed later.

### The MEF35 protein interacts with the mitochondrial MORF1 and MORF8 proteins

**Fig 3:**
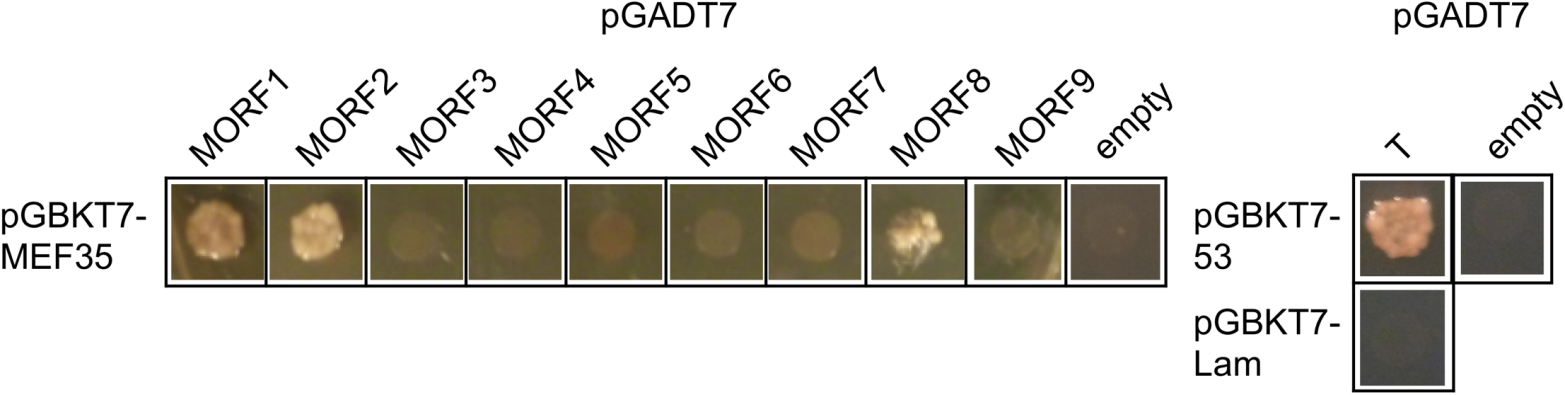
MEF35 interacts with the MORF1, MORF2 and MORF8 proteins. Yeast 2-hybrid (Y2H) assays reveal interactions with the mitochondrially located MORF1, the plastid MORF2 and the dual targeted MORF8. In a mutant of MORF1, the MEF35 target sites are not affected [22], some slight effects are seen upon knock-down in other assays [28]. In a respective MORF8 (also termed RIP1) mutant, editing at two of the three MEF35 target sites is reduced, suggesting that the interactions with MORF8 maybe functionally relevant. Controls include cotransfection of the pGBKT7-MEF35 construct with the pGADT7 vector without any MORF insert (empty) as well as the positive interaction control of murine p53 (pGBKT7-53) with the SV40 large T-antigen in pGADT7 (T) and no connection of human lamin C (pGBKT7-Lam) with the SV40 large T-antigen (T). The fusion protein pGBKT7-53 does not interact with the protein product of the empty pGADT7 vector (empty).

In addition to the E domain containing PPR proteins, MORF proteins are also required for effective RNA editing at most editing sites in plastids and mitochondria [22,28]. To investigate whether MORF proteins are also required at the MEF35 editing sites, we analysed their editing status in cDNAs of the respective *MORF* mutants. Among the three target sites of MEF35, *rpl16*-209 and *cob*-286 sites are reduced to about 70% in a *morf8*/*rip1* mutant while no significant reduction was detected in knock-down and T-DNA knock-out lines of other *MORF* genes (S2 Fig; [22,28]). We therefore investigated the potential of the MEF35 protein to interact with MORF8 and the other eight MORF proteins in yeast 2 hybrid (Y2H) assays (Fig 3) and observed interactions with MORF8, MORF1 and MORF2. Since MORF2 is exclusively plastid located [22, 28, 29], this connection is most likely due to the surface structure of the MORF2 protein which can contact many other plastid and mitochondrial PPR proteins besides MEF35 and should not be functionally relevant *in vivo*. MORF1 is exclusively mitochondrially located and MORF8 is dually targeted, suggesting that either or both connections may be important for processing the RNA editing target nucleotides of MEF35.

## Discussion

### The DYW subgroup PPR protein MEF35 is an RNA editing factor in mitochondria

The MEF35 protein is predicted by targetP ([26]) to contain a 24 amino acids long presequence which directs the protein to the mitochondria. RNA editing analysis confirms the mitochondrial location with the identification of three target sites in mitochondria. The loss of editing at the three identical nucleotides in two independent T-DNA insertion mutants and the recovery of editing by introduction of the gene for MEF35 into one of the mutant plant lines confirms the identification and functional assignment of MEF35 as a specific RNA editing factor in mitochondria.

### Prediction of RNA editing target sites for MEF35

We recently developed a prediction tool based on the specific connections between individual PPR elements and the contacted RNA sequence starting at nucleotide -4 and extending further upstream of the edited nucleotide [8]. The tool ranks targets by their matches and thus by probability. In the prediction compiled for the MEF35 protein, the *rpl16*-209 RNA editing site ranks first and site *nad4*-1373 is found at position 4 (Fig 2). The third site, *cob*-286 escaped from our prediction tool suggesting that the prediction tool requires further improvement. For all three MEF35 targets, even for site *cob*-286, the minimum of five PPR-nucleotide matches are identified with the prediction tool (Fig 2). This number of specific recognition matches includes the C-terminal L2 and S2 elements, which were considered to not contact the RNA by Barkan et al. [5]. However, several other editing sites, which also have five or more PPR-nucleotide matches with MEF35, are not affected in *mef35* mutants suggesting that only the number of PPR-nucleotide matches are not sufficient for the specific RNA editing site recognition. When we align the three MEF35 target sites, there are four shared PPR-nucleotide-matches at the -4, -5, -6 and -10 positions. We found four other mitochondrial RNA editing sites that share PPR-nucleotide matches to MEF35 targets at these four positions, *atp1*-1292, *atp4*-416, *rps12*-84 and *rps4*-226, which are all edited in the *mef35* mutants, suggesting that these four PPR-nucleotide-matches are not sufficient for the specific RNA recognition and that further PPR-nucleotide matches at least in *cob*-286 are involved, which are not detected or considered in the current prediction tool. For example, our present prediction algorithm does not take into account nucleotide–PPR combinations in which the amino acids at positions 6 and 1’ have no biased nucleotide correlations. This includes for example the two L-elements corresponding to the -8 and -12 positions of the MEF35 target editing sites. Other possibly hidden PPR-nucleotide matches are minor, i.e. rare, amino acid combinations at positions 6 and 1’. For instance, the P-element corresponding to the -13 nucleotide position before the editing site has amino acids A and D at position 6 and 1’, a combination which has previously been observed only once each in OTP87, CRR4 and CRR21 and aligns to U, G and G, respectively. This combination may also be involved in the biased PPR-nucleotide preference carrying the specificity of MEF35, since nucleotides at position -13 in all three MEF35 target sites are G. Alternatively, the clear five PPR-nucleotide matches could be sufficient for specific binding between MEF35 and the *cob*-286 RNA. In this case, interaction and binding in these five PPR-nucleotide connections must be stronger than other PPR-RNA connections at those RNA editing sites with five or more PPR-nucleotide matches that are not affected in the *mef35* mutants. Site *nad1*-571 at rank three by the prediction tool has six PPR-nucleotide matches with MEF35, but is addressed by editing factor MEF32 [8]. This site does not have a PPR-nucleotide match with MEF35 at the -10 position suggesting that binding at this position is more important than at other positions. Significant differences of PPR-nucleotide binding intensities have been observed between chloroplast RNA editing factor CLB19 and its target sequences [30]. These results suggest that each PPR carries different nucleotide binding intensity even with same amino acid combinations at position 6 and 1’ and imply a limitation of the current prediction tool which weighs all possible contact sites equally and does not consider binding intensities influenced by other amino acid differences in the respective PPR modules. These analyses suggest that the combinations between PPR elements and actually contacted nucleotides can be very flexible and yet maintain specificity.

### RNA editing at site *nad4*-1373 is independent of an upstream event

The next closest editing site to MEF35 target site *nad4*-1373 is located only 18 nucleotides upstream at site *nad4*-1355 and is fully edited in both mutants of MEF35 (Fig 1). This site *nad4*-1355 is the target of the E subgroup PPR protein MEF18 [31]. In a MEF18 T-DNA insertion mutant, editing at this site is lost, while editing at the MEF35 target site *nad4*-1373 is not affected. These observations show that both sites are processed independently and that there is no effect of the editing status at the upstream *nad4*-1355 on the editing capability of MEF35 at *nad4*-1373. It is therefore likely that the PPR elements of MEF35 bind the continuous sequence upstream of its target site *nad4*-1373. This binding sequence then ends at nucleotide -15, well before the upstream site at *nad4*-1355 (Fig 2). A mutant of MEF18 likewise has no detectable phenotype suggesting that neither of these RNA editing events is essential in the *Arabidopsis* plants.

### Amino acid change of the RNA editing event in RPL16

Since there is little detectable growth and developmental phenotype in the *mef35-1* mutant *Arabidopsis* plants (Fig 1C and S1 Fig), none of the affected RNA editing events can be essential. This is particularly surprising for the RPL16 protein, since loss of editing at another site, *rpl16*-458, in the maize mutant *empty pericarp5* (*emp5*) has very strong effects [32].

**Fig 4:**
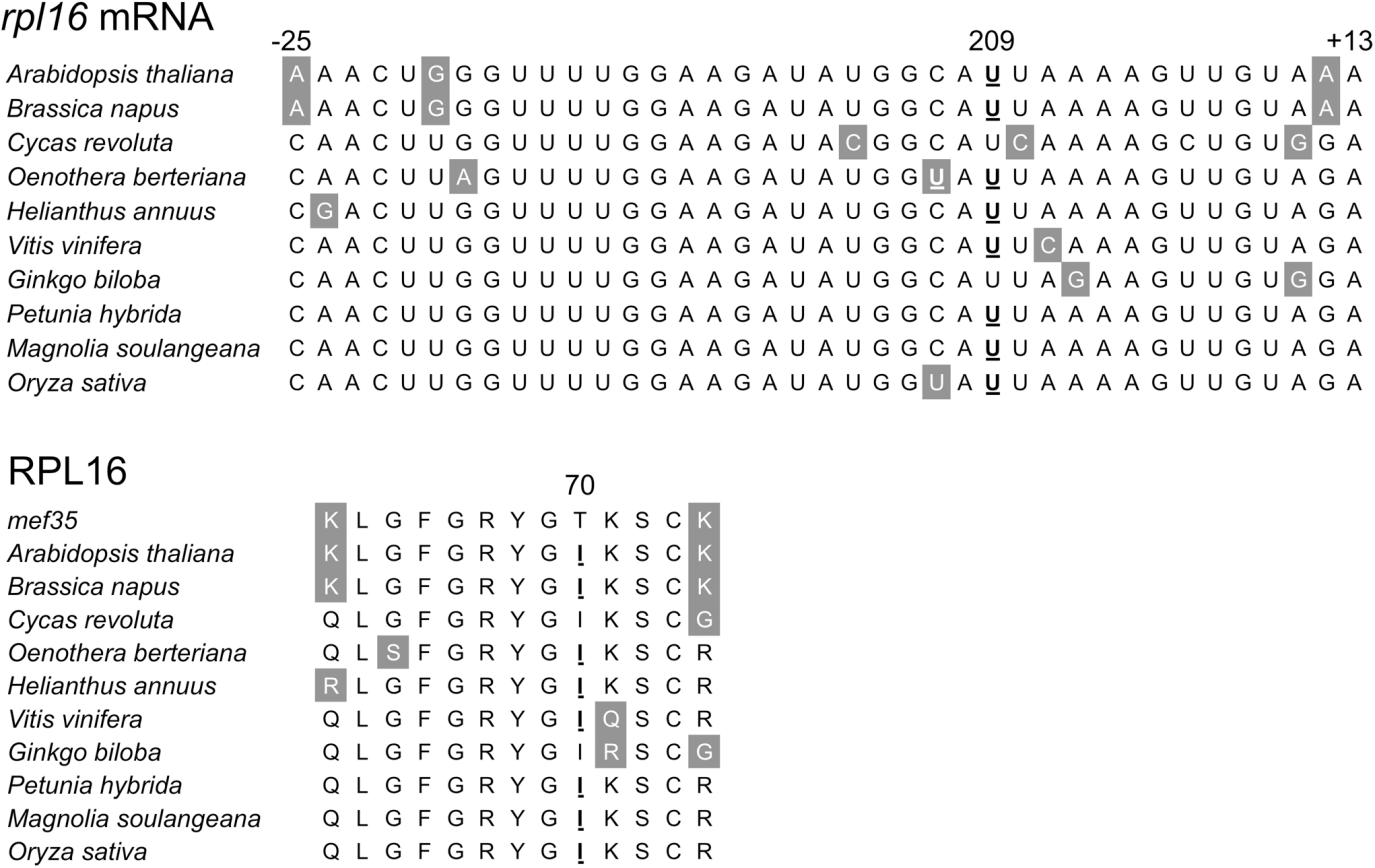
The *rpl16*-209 editing site is located in a highly conserved environment. **(A)** Comparison of nucleotide identities in the *cis*-recognition sequence around the *rpl16*-209 editing site with the homologous editing sites in other plant species reveals the high degree of conservation. This is presumably imposed by the functional constraints on the conservation of the amino acids surrounding the *rpl16*-209 editing site. Some of the plants compared here encode a genomic T at this position to maintain the amino acid identity. **(B)** The absence of RNA editing event *rpl16*-209 results in *Arabidopsis* in the incorporation of the genomically encoded threonine rather than the isoleucine specified by the edited codon number 70. The amino acid isoleucine is conserved in even distant plant species. Nucleotides and amino acids derived by RNA editing are given in bold letters and are underlined. Nucleotides and amino acids differing from the consensus are shown in inverse shading.

The amino acid sequence surrounding amino acid 70, which is altered from threonine to isoleucine by the MEF35 RNA editing event, is identical between various flowering plants, including rather distant species such as *Cycas* and *Gingko* (Fig 4). The gene for RPL16 is the only one for a ribosomal protein that is present in the mitochondrial genome of all land plants as well as algae, stramenopiles, alveolates and rhizaria [33]. Since such a high degree of conservation usually reflects a functional importance, it seems even more surprising that there is no obvious detectable phenotype in the *mef35-1* and *mef35-2* mutant plants.

In the *emp5* mutant in maize, loss of editing at site *rpl16*-458, results in embryo lethality [32]. This mutant confirms that the RPL16 protein is essential in the mitochondrial ribosome and thus for the plant. Previous analyses had shown that deletion of the mitochondrial *rpl16* gene is lethal and results in non-chromosomal stripes (mutant NCS3) in maize [34]. In *Arabidopsis*, a genomic rearrangement resulting in a disruption of the *rpl16* transcription unit by deletion of upstream sequences from the cotranscribed *rps3* gene leads to a severe phenotype [35]. It is therefore necessary to analyse the locations of the MEF35 mediated editing event and its potential influence on RPL16 function.

Proteins L16 and L27 are crucial components of the peptidyltransferase center in the 50S subunit of RPL16 and directly contact the A-site and the P-site, respectively [36]. Several antibiotics interact with the RPL16 protein which is assembled late into the ribosome [36–40]. Recent detailed analyses found that once incorporated, L5 and L16 facilitate tRNA translocation [41]. During translocation, the tRNAs are handed over from protein L16 to L5 and then to L1. The MEF35 *rpl16*-209 RNA editing site is located just before the main contact site with the tRNA during the translocation step when it is handed over to RPL5. The contacting fragments have the highest degree of conservation within the RPL16 protein and are especially sensitive to changes in the amino acid charges [40]. The MEF35 editing site is located outside of the main contact site, explaining why the amino acid change between isoleucine and threonine does not interfere with the tRNA connection for the tRNA handover and thus the function of RPL16.

### Interaction between the MEF35 and MORF1 and MORF8 proteins

Indirect evidence for a potential cooperation between a given PPR protein such as MEF35 and one of the MORF proteins can be provided by a comparison of editing at the target sites between mutants of the PPR protein and any or all of the nine full-sized MORF proteins. Such an analysis reveals that in mutants of MORF8, RNA editing is reduced to 70% at the *rpl16*-209 and *cob*-286 MEF35 target sites but is not affected at the *nad4*-1373 site [28]. None of the other MORF proteins shows an influence on RNA editing in the respective mutants (S2 Fig; [22,28].)

In the experimental analysis by yeast two-hybrid assays, the MEF35 PPR protein interacts with MORF1, MORF2 and MORF8 proteins. MORF2 is plastid located and therefore in a different organelle in *Arabidopsis thaliana* cells. The other two proteins, MORF1 and MORF8 are as MEF35 targeted to plant mitochondria. The relevance of the MEF35 –MORF1 interaction is not clear, but the MEF35 –MORF8 contact is potentially a functional connection since both proteins influence RNA editing at the same nucleotides.

It is unclear why only the editing at the *rpl16*-209 and *cob*-286 sites is affected in the MORF8 mutant. Similar distinct impact of the MORF8 mutation on the target sites of respective PPR RNA editing proteins has been observed also for CRR22, OTP81 MEF11 and COD1 targets. While one or few sites show little effect in a *morf8* mutant, other target sites of these RNA editing factors are strongly affected. For these RNA editing factors, MORF8 may support binding to RNA or to other co-factors such as other MORF, ORRM [42,43] or OZ [44] proteins, which are necessary for some target sites but not others. Extensive protein-protein interactions between the different MORF proteins has been reported recently [45]. The PPR protein MEF13 only weakly interacts with MORF3, but this connection is stabilized by the presence of MORF8 [46].

These analyses now contribute to a better understanding of the plant organellar editosomes and will allow further investigations. MEF35 offers a good platform to further analyse e.g. the MEF-MORF protein-protein interactions and their functional relevance.

## Materials and Methods

### Plant material and preparation of nucleic acids

Two T-DNA insertion lines of *Arabidopsis thaliana*, SAIL_101_B01 (*mef35-1*) and SALK_060464 (*mef35-2*), were obtained from the TAIR resources. *Arabidopsis thaliana* plants were grown in growth chambers under 21°C, 65% humidity, 16 h light and 8 h dark. Green leaves of about 1.5 cm length were harvested from the rosettes of about three week old plantlets. Preparation of DNA or RNA from the leaves was done as described [47]. In the mutants, T-DNA insertion sites were verified by PCR. Phenotypes of homozygous mutant plants were analysed in comparison to wild type Col plants grown alongside.

### Computational target prediction of At4g14050

Prediction of At4g14050 target sites was performed as described in Takenaka et al. [8]. The combination of the amino acids at positions 6 and 1’ of the individual eleven PPR elements were compared to the nucleotide most often associated with this set in the previously identified PPR elements to which target RNA sequences have been characterized. Some combinations of the 6th amino acid in several PPR elements and the first amino acids of the next element (1’) give a biased probability of the nucleotide most likely recognized by this respective PPR module. Each PPR thus has its own PPR-nucleotide connection probability profile. Using these profiles for the eleven PPR elements of MEF35, the probability to match the respective nucleotide bias is calculated for the putative binding sites of all editing sites in the coding regions of genes encoded in mitochondria of *Arabidopsis* by the FIMO program [48].

### Analysis of RNA editing sites

Relevant cDNA fragments covering one or more editing sites were obtained by RT-PCR amplification according to published protocols [47]. At the RNA editing sites, cDNA sequences were evaluated for the respective C to T differences. RNA editing levels were estimated by the relative areas of the respective nucleotide peaks in the sequence analyses. Ratios between areas were calculated with the DNA Dynamo program.

Sequences were obtained commercially from 4base lab, Reutlingen, Germany or from Macrogen, Seoul, Korea.

### Complementation assays in transgenic plants

Plants of the mutant line *mef35-2* were transformed with the *MEF35* wild type (wt) Col reading frame under control of the 35S promoter in vector pMDC123 [49] with a GFP cassette from psMGFP4 [50] and a multiple cloning site from pET41 (Merck Millipore Novagen®, Darmstadt, Germany) by floral dip [51] Transgenic plants of *mef35-2* were screened by spraying with Basta®. The respective RNA editing sites were analysed by direct sequencing of cDNA [47].

### Yeast two-hybrid analysis

The MEF35 and MORF protein coding sequences were cloned with the In-Fusion HD cloning system (Clontech Laboratories, Mountain View, USA) and transferred into the bait (pGBKT7) and prey (pGADT7) vectors of the GAL4 two Hybrid System 3 or the Matchmaker^TM^ Gold Yeast Two-Hybrid System (Clontech Laboratories, Mountain View, USA) for transformation into yeast cells (PJ69-4A) [45,52–54]. For cloning, the mitochondrial target sequence was excluded to not interfere with the interaction studies. Yeast cells grown on medium without leucine and tryptophan were selected for transfection of the plasmids. Interaction assays were done by dropping 5 μl of an overnight liquid culture adjusted to OD-_600nm_ of 0.1 and in addition 0.3 [45] in SD medium lacking T, L, H, A (synthetic dropout, Clontech Laboratories, Mountain View, USA) to the agar medium plate. For stringent selection, drop tests on the same medium but with 2.5 mM 3AT added were assayed in parallel. All assays were done at least twice.

## Supporting Information

S1 Fig Adult plants of mutant line *mef35-2* are indistinguishable from wild type plants. While seedlings of mutant line *mef35-2* initially develop a little slower in comparison to the wild type Col, adult plants at the flowering stage after seven weeks on soil look very similar.
(PDF)

S2 Fig Editing at the MEF35 target sites is not affected in mutants of five mitochondrial MORF proteins. Percentages of RNA editing at the target sites are shown as determined by direct sequence analysis. As this experimental approach does allow to not distinguish between a background of up to 10% and genuine effects, values above 90% have to be considered as full editing. These data are experimentally determined from the mutant lines *morf1-1* EMS mutant, *morf3-1* (GK-109E12.01), *morf4-1* (SAIL_731_D08), *morf5-1* (SALK_016801C), *morf6-1* (GK-184F04.01).
(PDF)
