# Supplementary material for: The DYW Subgroup PPR Protein MEF35 Targets RNA Editing Sites in the Mitochondrial rpl16, nad4 and cob mRNAs in Arabidopsis thaliana
Source: PLoS One. 2015 Oct 15;10(10):e0140680. doi: 10.1371/journal.pone.0140680 (PMC4607164; doi:10.1371/journal.pone.0140680)
Supplement: S1 Fig — While seedlings of mutant line mef35-2 initially develop a little slower in comparison to the wild type Col, adult plants at the flowering stage after seven weeks on soil look very similar. (PDF) [file pone.0140680.s001.pdf]

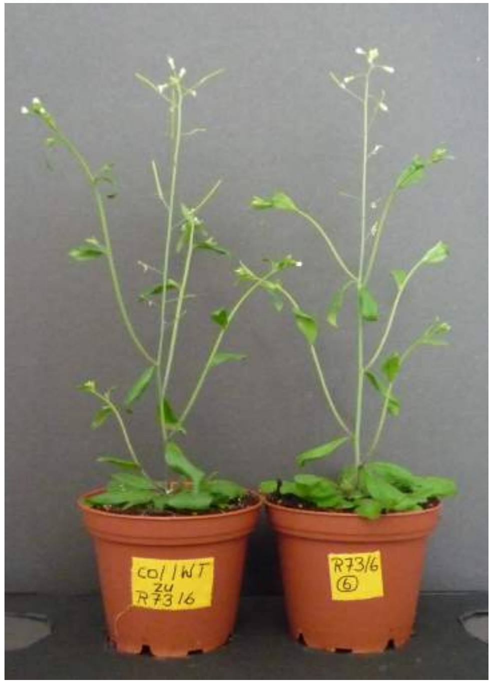

Col *mef35-2*

Fig S1 Adult plants of mutant line *mef35-2* are indistinguishable from wild type plants. While seedlings of mutant line *mef35-2* initially develop a little slower in comparison to the wild type Col, adult plants at the flowering stage after seven weeks on soil look very similar.

.
