# Supplementary material for: The DYW Subgroup PPR Protein MEF35 Targets RNA Editing Sites in the Mitochondrial rpl16, nad4 and cob mRNAs in Arabidopsis thaliana
Source: PLoS One. 2015 Oct 15;10(10):e0140680. doi: 10.1371/journal.pone.0140680 (PMC4607164; doi:10.1371/journal.pone.0140680)
Supplement: S2 Fig — Percentages of RNA editing at the target sites are shown as determined by direct sequence analysis. As this experimental approach does allow to not distinguish between a background of up to 10% and genuine effects, values above 90% have to be considered as full editing. These data are experimentally determined from the mutant lines morf1-1 EMS mutant, morf3-1 (GK-109E12.01), morf4-1 (SAIL_731_D08), morf5-1 (SALK_016801C), morf6-1 (GK-184F04.01). (PDF) [file pone.0140680.s002.pdf]

gene	editing site	Col	*morf1-1*	*morf3-1*	*morf4-1*	*morf5-1*	*morf6-1*
cob	286	98	96	97	96	99	98
rpl16	209	99	94	99	96	100	99
nad4	1373	92	92	92	99		99

Fig S2 Editing at the MEF35 target sites is not affected in mutants of five mitochondrial MORF proteins. Percentages of RNA editing at the target sites are shown as determined by direct sequence analysis. As this experimental approach does allow to not distinguish between a background of up to 10% and genuine effects, values above 90% have to be considered as full editing. These data are experimentally determined from the mutant lines *morf1-1* EMS mutant, *morf3-1* (GK-109E12.01), *morf4-1* (SAIL_731_D08), *morf5-1* (SALK_016801C), *morf6-1* (GK-184F04.01).
